# Development and characterization of liposomal formulations containing sesquiterpene lactones for the treatment of chronic gout

**DOI:** 10.1038/s41598-024-57663-1

**Published:** 2024-03-24

**Authors:** Rafaela Cunha Matosinhos, Frédéric Frézard, Sabrina Mendes Silva Araújo, Andressa Magalhães Barbosa, Isabela Fernanda de Souza, José Dias de Souza Filho, Jacqueline de Souza, Ana Paula Corrêa Oliveira Bahia, Francesca Ietta, Agnese Magnani, Dênia Antunes Saúde-Guimarães

**Affiliations:** 1 Laboratório de Plantas Medicinais (LAPLAMED), Programa de Pós-Graduação em Ciências Farmacêuticas (CiPharma), Escola de Farmácia, Universidade Federal de Ouro Preto, Ouro Preto, Minas Gerais 35400-000 Brazil; 2 Laboratório de Biofísica e Sistemas Nanoestruturados (LabNano), Departamento de Fisiologia e Biofísica, Instituto de Ciências Biológicas, Universidade Federal de Minas Gerais (UFMG), Belo Horizonte, Minas Gerais 31270-901 Brazil; 3 Laboratório Multiusuário de Caracterização de Moléculas (LMCM), Escola de Farmácia, Universidade Federal de Ouro Preto, Ouro Preto, Minas Gerais 35400-000 Brazil; 4 Laboratório de Controle de Qualidade de Insumos, Fármacos e Medicamentos (LCQ), Escola de Farmácia, Universidade Federal de Ouro Preto, Ouro Preto, Minas Gerais 35400-000 Brazil; 5 Dipartimento Scienze della Vita, Università degli Studi di Siena, 53100 Siena, Tuscany Italy; 6 Dipartimento di Biotecnologie, Chimica e Farmacia, Università degli Studi di Siena, 53100 Siena, Tuscany Italy

**Keywords:** Rheumatology, Rheumatic diseases, Biochemistry, Enzymes, Acute inflammatory arthritis, Crystal deposition arthropathies, Nanoscience and technology, Nanomedicine, Drug delivery

## Abstract

Gout and hyperuricemia are characterized by high uric acid levels, and their treatment involves medications that have adverse effects. In this study, we evaluated oral liposomal formulations with eremantholide C and goyazensolide as a novel approach to reduce the toxicity associated with these substances while maintaining their anti-hyperuricemic activity. We characterized the formulations and evaluated them based on encapsulation efficiency and stability over 12 months and under simulated physiological environments. We determined the toxicity of the liposomal formulations in Caco-2 cells and the anti-hyperuricemic activity in rats. The formulations exhibited nanometric size, a narrow size distribution, and a negative zeta potential, indicating their stability and uniformity. The efficient encapsulation of the sesquiterpene lactones within the liposomes emphasizes their potential for sustained release and therapeutic efficacy. Stability evaluation revealed a small decrease in the eremantholide C concentration and a remarkable stability in the goyazensolide concentration. In Caco-2 cells, the liposomes did not exert toxicity, but did exhibit an antiproliferative effect. In vivo assays demonstrated that the liposomes reduced serum uric acid levels. Our study represents an advancement in gout and hyperuricemia treatment. The liposomal formulations effectively reduced the toxicity associated with the sesquiterpene lactones while maintaining their therapeutic effects.

## Introduction

Hyperuricemia is characterized by serum uric acid (UA) levels higher than 7.06 mg/dL (in men) or 6.05 mg/dL (in women). It is the most important risk factor for gout, as it creates an environment conducive to the formation and deposition of urate crystals^1,2^. Gout is a form of inflammatory arthritis characterized by the deposition of urate crystals in the joints, which leads to recurrent episodes of intense pain and inflammation. The approximate occurrence is 0.2–0.35 affected people per 100 inhabitants of the world population^1,3^. The current gout therapeutic approaches focus on managing acute attacks and preventing future occurrences^4,5^. Nonsteroidal anti-inflammatory drugs (NSAIDs), colchicine, and corticosteroids are commonly used to alleviate pain and to reduce inflammation during acute episodes. Additionally, urate-lowering drugs (ULDs), such as xanthine oxidase (XO) inhibitors (e.g., allopurinol and febuxostat) and uricosuric agents (e.g., benzbromarone and probenecid), are used to reduce urate production or to increase UA excretion. In this way, they are able to decrease serum urate levels and to prevent crystal formation, thereby reducing hyperuricemia and the frequency of gout attacks. ULDs may be associated with skin reactions, hepatic side effects, changes in liver function tests, and undesirable cardiovascular effects^1,6–11^. Furthermore, adherence to ULD treatment is the lowest when it comes to chronic diseases^1^. Therefore, it is imperative for ongoing research in gout therapy to identify better-tolerated interventions that are capable of enhancing efficacy, minimizing adverse effects, and improving patient outcomes.

**Figure 1:**
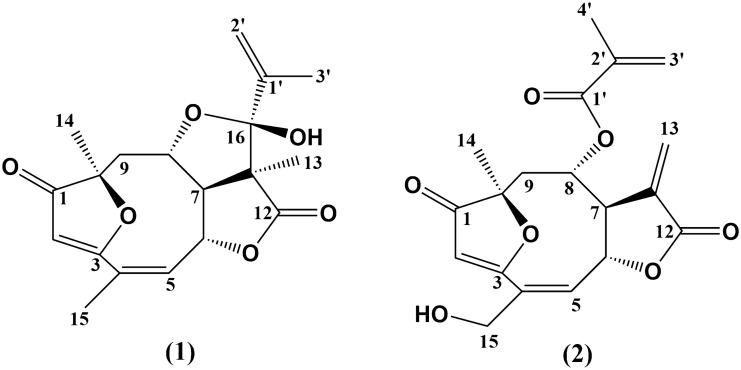
Chemical structure of the sesquiterpene lactones eremantholide C (1) and goyazensolide (2).

In the ever-evolving landscape of gout and hyperuricemia management, the search for new and better-tolerated therapies has spurred a focused exploration into natural compounds, particularly sesquiterpene lactones^12,13^. Among these, eremantholide C (EREC) and goyazensolide (GOIA), whose structures are shown in Fig. 1, have emerged as promising candidates, showing the potential to modulate hyperuricemia, inflammation, pain, and oxidative stress, and to mitigate gout symptoms^14–16^. As described by Bernardes et al.^14,15^, these two sesquiterpene lactones are capable of modulating the inflammation in gout by inhibiting neutrophil migration and the release of pro-inflammatory cytokines and are capable of tempering hyperuricemia by reducing serum UA levels through an inhibition of XO activity and an increase in UA excretion. However, their therapeutic effects are moderated by the presence of functional groups that could lead toxicity, particularly due to the presence of the α-methylene-γ-lactone group and methacrylate ester (cyclic or acyclic), which have electrophilic properties, making them prone to interact with cellular nucleophiles^17,18^. Furthermore, these sesquiterpene lactones are lipophilic. Biopharmaceutical studies have shown that EREC has high membrane permeability, but low solubility and some instability under physiological environments^19^. GOIA presented high solubility, and adequate stability under physiological conditions, however, it presented cytotoxicity^20^.

In this context, nanostructured formulations emerge as an alternative for carrying lipophilic substances, enhancing their water solubility, and stability under physiological conditions. Liposomes are vesicles composed of lipid bilayers, capable of encapsulating hydrophilic drugs and incorporating lipophilic substances such as the sesquiterpene lactones. Thus, liposomal formulations are well established for their ability to improve oral drug bioavailability and decrease drug toxicity, by increasing drug solubility and stability and reducing cytotoxicity^21,22^. Therefore, we decided to formulate the sesquiterpene lactones EREC and GOIA within liposomes as a strategic approach to increase their stability and solubility, minimizing the risk of the toxicity associated with these substances, while preserving their anti-gout and anti-hyperuricemic effects.

## Results

### Characterization of the liposomal formulations

Two liposomal formulations were prepared from soybean phosphatidylcholine (SPC) containing EREC and GOIA (LIPO + EREC and LIPO + GOIA, respectively), both at lipid/substance molar ratio of 20:1. Empty (substance-free) liposomes (LIPO) were also prepared. They were characterized in relation to the particle hydrodynamic diameter, polydispersity index (PDI), and zeta potential (ζ) with a Zetasizer. Furthermore, the encapsulation efficiencies of the substances (i.e., the EE) was investigated. The results are presented in Table 1.

**Table 1:** Characterization of the liposomal formulations containing the sesquiterpene lactones.

Liposomal formulation	Size (nm ± S.E.M)	PDI (value ± S.E.M)	Zeta potential (mV ± S.E.M)	Concentration of the substance (µg/mL ± S.E.M)	EE (% ± S.E.M)
LIPO	110.9 ± 6.25	0.086 ± 0.031	−5.11 ± 1.55	–	–
LIPO + EREC	108.5 ± 7.92	0.053 ± 0.026	−4.42 ± 0.10	27.5 ± 6.50	69.79 ± 3.55
LIPO + GOIA	111.5 ± 2.29	0.049 ± 0.014	−4.68 ± 0.62	42.00 ± 5.00	79.62 ± 6.42

In all developed formulations, unimodal and narrow particle size distributions were evidenced by PDI values less than 0.1 (0.086 ± 0.031, 0.053 ± 0.033, and 0.049 ± 0.013 for LIPO, LIPO + EREC and LIPO + GOIA, respectively). All formulations presented nanometric size with diameter around 110 nm (110.9 ± 6.25 nm, 108.5 ± 7.92 nm, and 111.5 ± 2.29 nm for LIPO, LIPO + EREC and LIPO + GOIA, respectively), as expected from the size calibration step using 100-nm polycarbonate membranes. There was no significant difference between the size or the PDI of the liposomal formulations containing the sesquiterpene lactones (LIPO + EREC and LIPO + GOIA), when compared with the substance-free liposomes (LIPO). Furthermore, no significant change in mean diameter, PDI and zeta-potential was observed, when the stability of LIPO was evaluated under temperature stress, supporting colloidal stability of the liposome suspension under these conditions (see Supplementary Fig. S1).

Both liposomal formulations had a slightly negative zeta potential (−4.42 ± 0.10 mV and −4.68 ± 0.62 mV for LIPO + EREC and LIPO + GOIA, respectively), as did the substance-free liposome (LIPO; −5.11 ± 1.55 mV), without a significant difference between them. The low zeta potential of the liposomes, close to zero, can be attributed to the zwitterionic character of phosphatidylcholine, which is present at 90% in SPC. The lack of significant influence of EREC and GOIA incorporation on the surface potential of the liposomes was expected because of the non-ionizable state of these substances. LIPO + EREC presented an EREC concentration of 27.5 ± 6.50 µg/mL and an EE close to 70%, which indicates a significant amount of this sesquiterpene lactone was encapsulated successfully within the liposome. This result may be explained by the fact that EREC has a lipophilic nature that allows an effective incorporation into the liposomal membrane. EREC’s hydrophobic regions may facilitate interactions with the hydrophobic tails of phospholipids, promoting encapsulation.

LIPO + GOIA presented a GOIA concentration of 42.00 ± 5.00 µg/mL and an EE close to 80%, indicating that a substantial amount of GOIA was encapsulated successfully within the liposomes. The lipophilic functional groups in GOIA’s structure may contribute to interactions with the lipid bilayer, which facilitates its incorporation into the liposomal membrane.

Furthermore, the evaluation of the encapsulation efficiency of calcein as a fluorescent hydrophilic marker showed the existence of an internal aqueous compartment in our developed formulations, consistent with the formation of lipid vesicles, indicating that it is also possible to co-encapsulate a hydrophilic substance in liposomes (see Supplementary Fig. S2). Both developed formulations encapsulated calcein, but the encapsulation efficiency of the formulation with EREC (LIPO + EREC, 9%) was similar to that of empty liposomes (LIPO) and lower than that of the formulation with GOIA (LIPO + GOIA, 18%), as shown in Supplementary Fig. S2.

## Evaluation of the stability of the liposomal formulations

**Figure 2:**
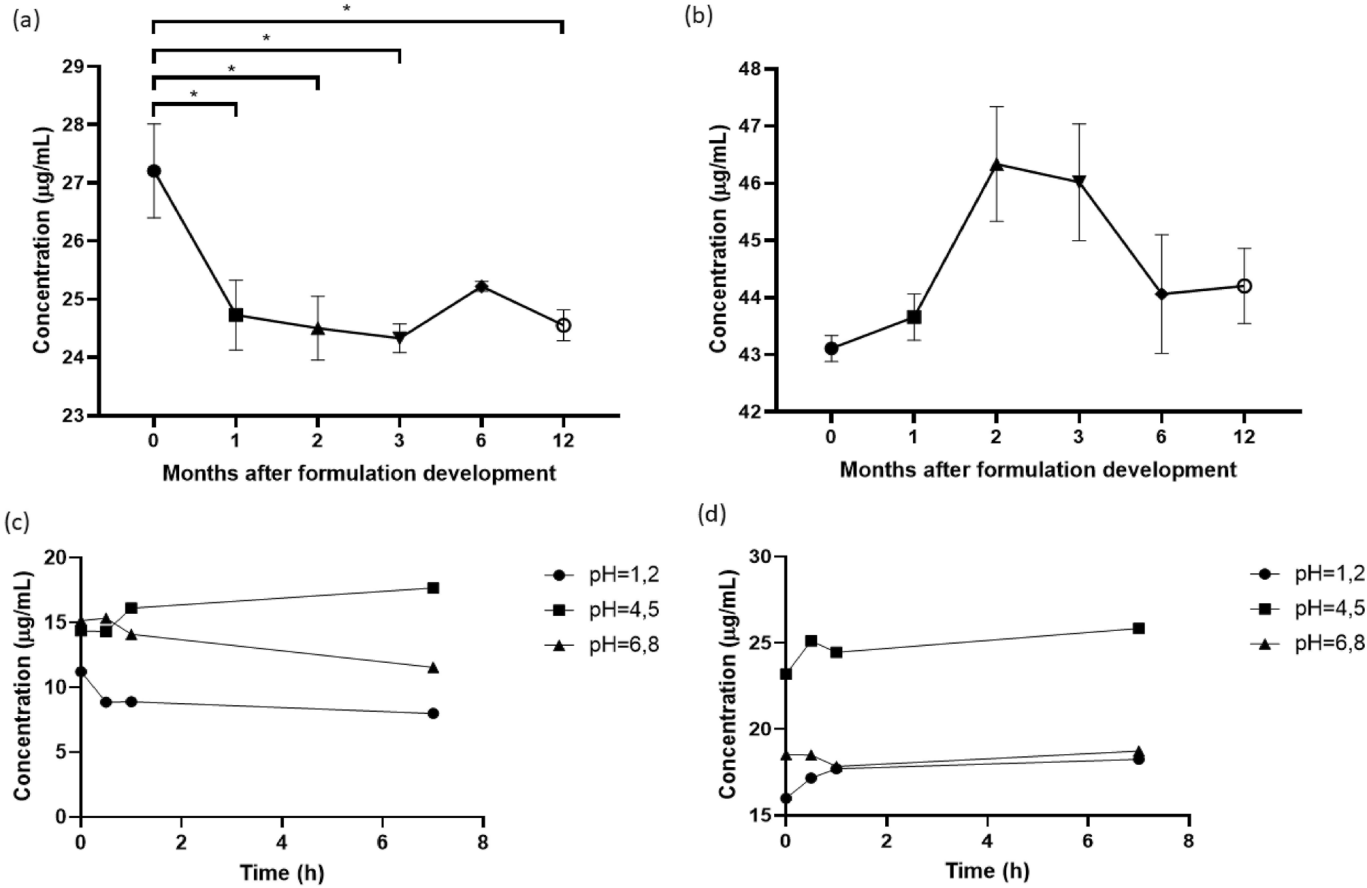
Stability over 12 months of the liposomal formulations LIPO + EREC (**a**) and LIPO + GOIA (**b**). Stability under simulated physiological environments among 7 h of the liposomal formulations LIPO + EREC (**c**) and LIPO + GOIA (**d**). *LIPO + EREC* liposomal SPC formulation containing eremantholide C; *LIPO + GOIA* liposomal SPC formulation containing goyazensolide. Values were expressed as mean ± S.E.M. One-way ANOVA was used followed by Tukey’s test for statistical significance (**a,b**). Two-way ANOVA was used followed by Tukey's multiple comparisions test for statistical significance (**c,d**). *P < 0.05 for multiple comparisions. Results show that the developed liposomal formulations are stable up to 12 months, and that the stability of the liposomal formulations is a little influenced by the pH.

The results of the stability evaluation of our two liposomal formulations containing the sesquiterpene lactones, after storage at 4°C in a refrigerator, are shown in Fig. 2a, b. They were obtained through spectrophotometric quantification at various time points: 1 week (i.e., 0 months) and 1, 2, 3, 6, and 12 months post-formulation development. We examined the stability of these formulations over an extended period to assess their robustness and potential for sustained therapeutic efficacy.

Furthermore, we evaluated the stability of the liposomal formulations containing EREC and GOIA, designed for oral administration by using HPLC within simulated physiological environments, including gastric fluid without enzymes (pH 1.2), acetate buffer (pH 4.5), and simulated intestinal fluid without enzymes (pH 6.8). The significance of this analysis stems from the intended oral delivery of the fomulations. Thus, it is necessary to understand their behavior under fluids that mimic the gastrointestinal tract. The results are shown in Fig. 2c, d.

As shown in Fig. 2a, compared with the EREC concentration at 1 week post-formulation development, LIPO + EREC exhibited a substantial decrease of 9.11%, 9.96%, 10.58%, and 9.74% at 1, 2, 3, and 12 months, respectively. At the initial time point (0 months), the concentration was 27.21 ± 0.81 µg/mL, and subsequent time points demonstrated a stable trend in the concentration, without a statistically significant difference between the time points. Specifically, the concentrations at 1, 2, 3, 6, and 12 months post-formulation development were 24.73 ± 0.60 µg/mL, 24.50 ± 0.55 µg/mL, 24.33 ± 0.25 µg/mL, 25.22 ± 0.086 µg/mL, and 24.56 ± 0.26 µg/mL, respectively.

LIPO + GOIA was able to maintain the initial GOIA concentration up to 12 months (Fig. 2b). There was no significant difference in the GOIA concentration at the evaluated times. The concentrations were 43.11 ± 0.23 µg/mL, 43.66 ± 0.41 µg/mL, 46.34 ± 1.00 µg/mL, 46.02 ± 1.02 µg/mL, 44.06 ± 1.04 µg/mL, and 44.20 ± 0.66 µg/mL for 1 week and 1, 2, 3, 6, and 12 months post-formulation development, respectively.

As shown in Fig. 2c, the initial concentration (0 h) of EREC within the LIPO + EREC was 11.23, 14.38, and 15.15 µg/mL at a pH of 1.2, 4.5, and 6.8, respectively. After 30 min, there was a significant decrease in concentration to 8.87 µg/mL (21.02%) at pH 1.2, which may suggest the beggining of some degradation or instability in the acidic gastric environment. This result was expected because, as described by Caldeira et al.^19^, EREC is less stable in acidic pHs due to the hydrolysis of lactones in acidic environments. Furthermore, an in vitro release test of EREC from the liposomal formulation showed a half-life of 3.7 h, which is shorter that the one exhibited by GOIA (4.9h) (see Supplementary Fig. S3). At pH 4.5, there was a slight decrease in concentration to 14.32 µg/mL (0.42%). At pH 6.8, the concentration increased to 15.35 µg/mL (1.32%), indicating a potential response to the less acidic environment. After 1 h, at pH 1.2, the EREC concentration remained low (8.9 µg/mL), suggesting the sustained impact of gastric conditions. At pH 4.5, the concentration increased to 12.57% (16.12 µg/mL), indicating some potential release or some change to the compound in the buffer. At pH 6.8, there was an 8.08% decrease (14.11 µg/mL), indicating a small change in stability. After 7 h, at pH 1.2, the EREC concentration decreased to 8.01 µg/mL (10.0%), indicating the impact of gastric conditions. At pH 4.5, there was a significant increase to 17.67 µg/mL (9.62%). Finally, at pH 6.8, the EREC concentration decreased to 11.55 µg/mL (18.14%). This decrease may be related to EREC release from liposomes (half-life of 3.7 h, as shown in Supplementary Fig. S3).

As shown in Fig. 2d, the initial concentration (0 h) of GOIA within LIPO + GOIA was 15.99, 23.19, and 18.53 µg/mL at pH 1.2, 4.5, and 6.8, respectively. After 30 min, there was a significant increase in the concentration to 17.17 µg/mL (7.38%) at pH 1.2, which may suggest some potential changes in the liposomal formulation in the gastric environment. At pH 4.5, there was another increase in concentration to 25.12 µg/mL (8.32%), which may indicate a potential release or alteration of GOIA in the acetate buffer. At pH 6.8, the concentration increased to 25.12 µg/mL (35.56%), indicating a potential response to the less acidic environment. After 1 h, at pH 1.2, the GOIA concentration increased to 17.71 µg/mL (3.15%). At pH 4.5, the concentration decreased to 24.46 µg/mL (2.63%). At pH 6.8, there was a 3.57% decrease (17.84 µg/mL), indicating a small change in stability. After 7 h, at pH 1.2, the GOIA concentration continued to increase to 18.26 µg/mL (3.11%), indicating stabilization under gastric conditions. At pH 4.5, there was a significant increase to 25.84 µg/mL (5.64%). At pH 6.8, the GOIA concentration increased to 18.73 µg/mL (4.99%). The higher stability of LIPO + GOIA may be related to the presence of the α-methylene-γ-lactone group in its structure, which stabilizes the molecule by resonance, leading to a longer half-life (4.9h) and a slower substance release (see Supplementary Fig. S3).

### Cell viability

**Figure 3:**
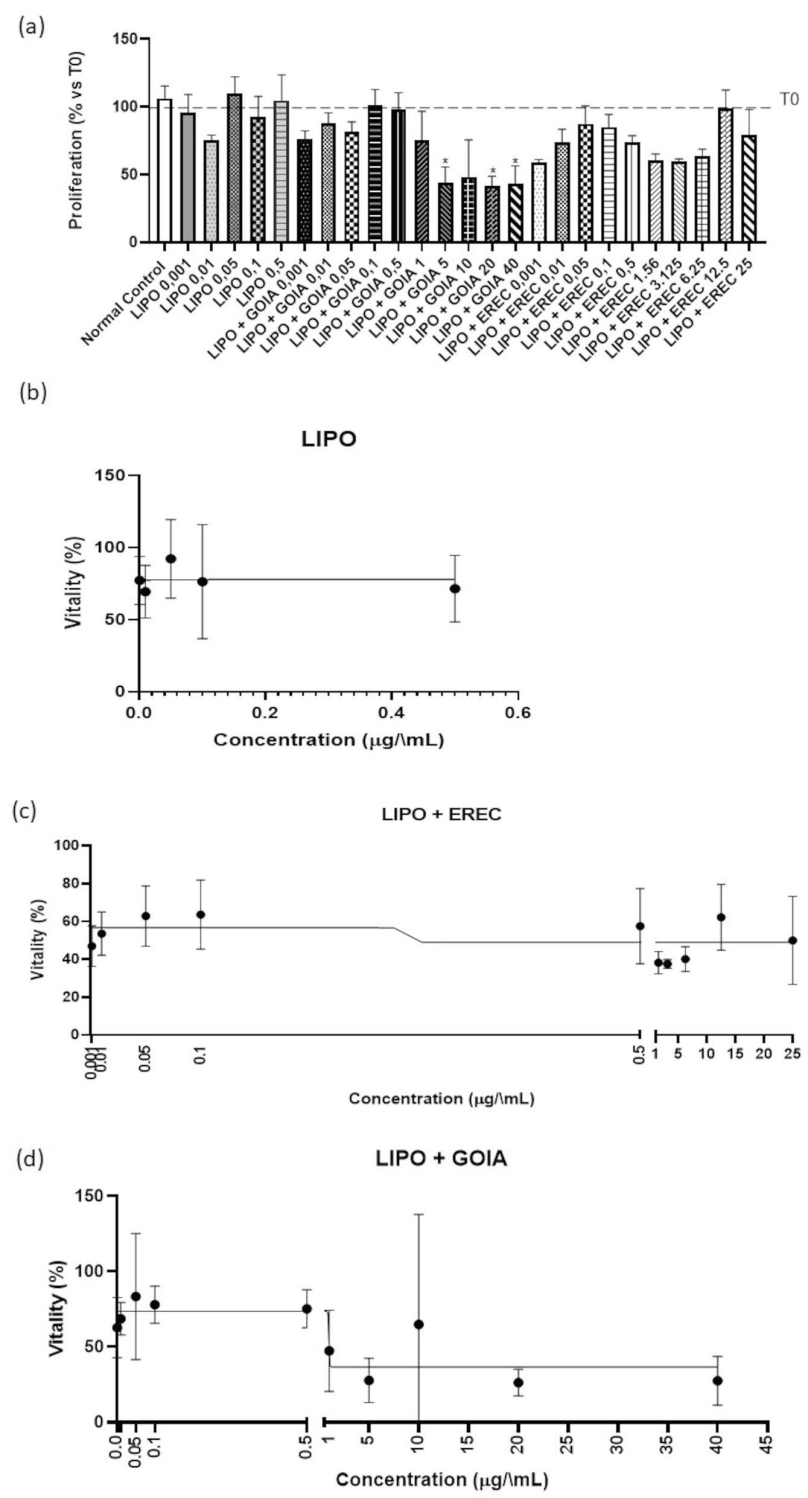
Caco-2 cells proliferation (**a**) and vitality (**b–d**) obtained from the sulforodhamine B assay. Normal control = DMEM, 10% FBS and 1% l-glutamin; *LIPO* substance-free liposome; *LIPO + EREC* liposomal SPC formulation containing eremantholide C; *LIPO + GOIA* liposomal SPC formulation containing goyazensolide. Values were expressed as mean ± S.E.M. One-way ANOVA was used followed by Dunnett’s test for statistical significance. *P < 0.05 compared with the normal control group. The results reveal intriguing insights of the impact of liposomal formulations containing the sesquiterpene lactones in cell vitality.

We tested the liposomal formulations containing EREC and GOIA and the substance-free liposome (LIPO) in Caco-2 cells, a well-established in vitro model for the intestinal epithelium, by using the SRB assay to evaluate the potential of these formulations to modulate cell proliferation and vitality. The results are shown in Fig. 3.

In Fig. 3a, we considered the initial cell counts at T0 to be 100%, providing a baseline for the proliferation results. We carefully chose the tested concentrations for each treatment to span a range that could allow a detailed assessment of dose-dependent effects. We used concentrations from 0.001 to 0.5 µg/mL for LIPO; it presented a half maximal inhibitory concentration (IC_50_) of 1.07E−16 (Fig. 3b). This exceptionally low IC_50_ indicates that LIPO had no toxic effect on Caco-2 and no effect on cell proliferation. In comparison, treatment with LIPO + EREC at 0.001–25 µg/mL produced an IC_50_ of 0.2798 (Fig. 3c). Treatment with LIPO + GOIA at 0.001–40 µg/mL produced an IC_50_ of 0.9645 (Fig. 3d). Treatment with LIPO + GOIA significantly reduced Caco-2 proliferation at concentrations of 5, 20, and 40 µg/mL, namely 44.11% ± 11.73%, 41.88% ± 7.053%, and 43.75% ± 12.87%, respectively (Fig. 3a), which may indicate an interference in the signaling pathways involved in cell cycle progression. By significantly reducing cell proliferation, LIPO + GOIA may indirectly modulate inflammatory responses associated with hyperuricemia and gout. Further studies should consider the mechanisms of how this modulation may occur.

### UA quantification and XO activity

**Figure 4:**
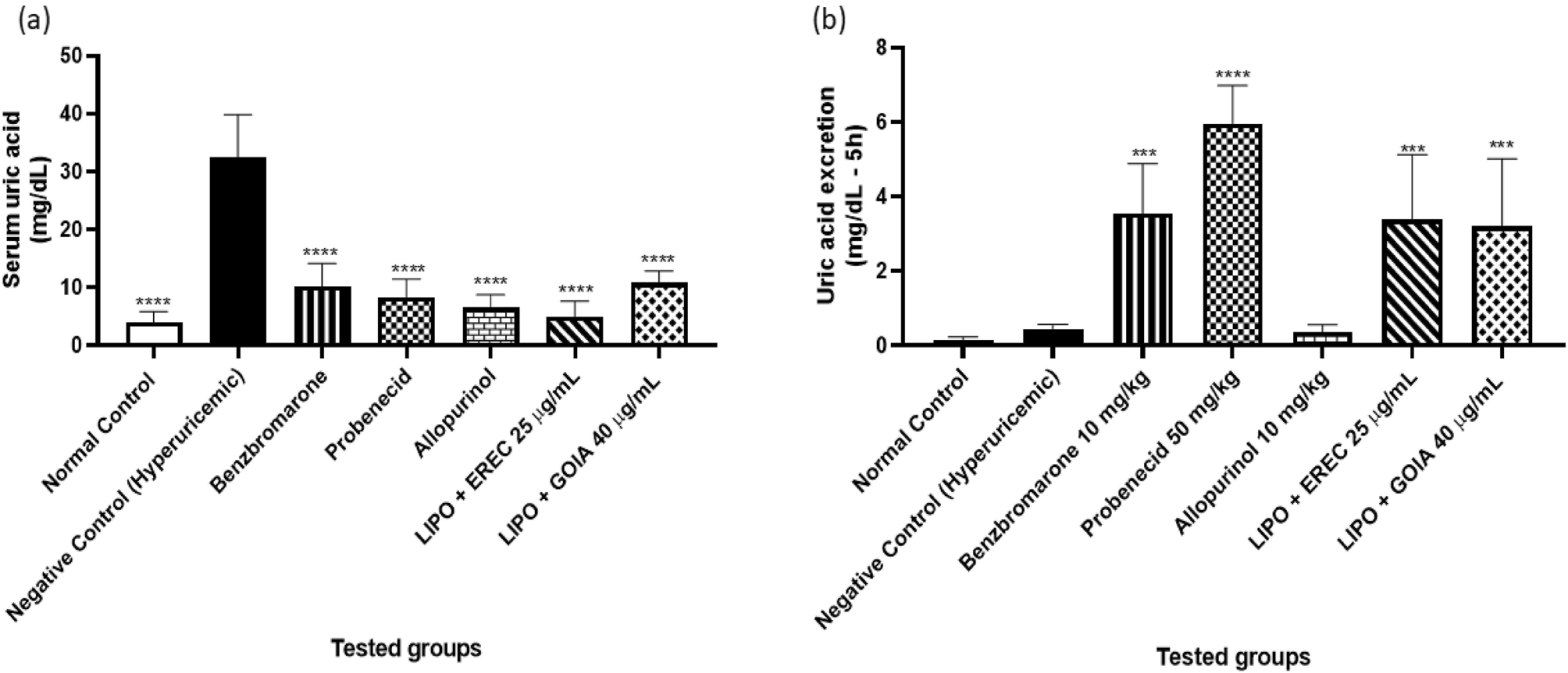
Serum uric acid levels (**a**) and uric acid excretion (**b**) after treatment of hyperuricemic rats with clinical used drugs and liposomal formulations containing sesquiterpene lactones. *LIPO + EREC* liposomal SPC formulation containing eremantholide C; *LIPO + GOIA* liposomal SPC formulation containing goyazensolide. Values were expressed as mean ± S.E.M. One-way ANOVA was used followed by Dunnett's test for statistical significance. ***P < 0.001 compared with the negative control group (hyperuricemic group); ****P < 0.0001 compared with the negative control group (hyperuricemic group). Results show that our developed formulations are capable of reducing serum uric acid levels in animals induced to hyperuricemia.

We successfully induced hyperuricemia in rats by administering potassium oxonate and UA. As shown in Fig. 4a, the hyperuricemic group presented a serum UA concentration of 34.31 ± 3.38 mg/dL, a significant increase compared with the normal control group (3.95 ± 0.74 mg/dL) (Table 2), probably due to the intense activity of the XO in the negative control group (25.10 ± 1.75 nmol/min/mg protein; Table 2). Following hyperuricemia induction, we administered treatments including uricosuric controls (benzbromarone and probenecid) for the UA quantification assay and an XO inhibitor (allopurinol) for the XO quantification assay and treated the experimental groups with LIPO + EREC or LIPO + GOIA. We quantified UA in urine and blood samples and XO activity in liver samples. The results are shown in Fig. 4.

**Table 2:** Effects of the liposomal formulations with eremantholide C (EREC) and goyazensolide (GOIA) and clinical used drugs on serum uric acid levels and xanthine oxidase activity in rats induced to hyperuricemia.

Group	Dose	Water intake (mL ± S.E.M.)	Urine volume (mL ± S.E.M.)	Uric acid excretion (mg/dL 5 h ± S.E.M.)	Serum uric acid (mg/dL ± S.E.M.)	Xanthine oxidase activity (nmol/min/mg protein ± S.E.M.)	Inhibition (%)
Normal control	–	6.25 ± 0.49	3.15 ± 0.60^d^	0.14 ± 0.03	3.95 ± 0.74^d^	6.28 ± 1.11^d^	74.98^d^
Negative control (hyperuricemic)	–	6.01 ± 1.21	6.33 ± 0.92	0.44 ± 0.052	34.31 ± 3.38	25.10 ± 1.75	–
Benzbromarone	10 mg/kg	10.51 ± 1.52^d^	9.43 ± 1.02^d^	3.55 ± 0.55^d^	10.13 ± 1.82^d^	–	–
Probenecid	50 mg/kg	8.00 ± 2.16^c^	8.35 ± 2.15^c^	5.95 ± 0.39^d^	8.36 ± 1.27^d^	–	–
Allopurinol	10 mg/kg	5.75 ± 1.67	6.99 ± 1.08	0.36 ± 0.086	6.62 ± 0.87^d^	8.66 ± 1.34^d^	65.51^d^
LIPO + EREC	25 µg/mL	5.19 ± 0.31	7.09 ± 0.38^b^	3.34 ± 0.65^d^	5.02 ± 0.93^d^	25.06 ± 2.14	–
LIPO + GOIA	40 µg/mL	6.44 ± 0.64	7.79 ± 0.34^b^	3.19 ± 0.78^d^	10.80 ± 0.85^d^	23.68 ± 3.34	–

Values were expressed as mean ± S.E.M. of eight animals. One-way ANOVA was used followed by Dunnett's test for statistical significance.

^a^P < 0.05 compared with the hyperuricemic control group.

^b^P < 0.01 compared with the hyperuricemic control group.

^c^P < 0.001 compared with the hyperuricemic control group.

^d^P < 0.0001 compared with the hyperuricemic control group.

As shown in Fig. 4a and Table 2, treatment with 10 mg/kg allopurinol reduced the serum UA level to 6.62 ± 0.87 mg/dL (80.71%), but as expected, it had no effect on UA excretion (Fig. 4b). Allopurinol is a known XO inhibitor; thus, the UA reduction induced by allopurinol may be explained by its reduction of XO activity (65.51%, Table 2). Treatments with uricosuric drugs, benzbromarone and probenecid, reduced the UA level to 10.13 ± 1.82 mg/dL (70.48%) and 8.36 ± 1.27 mg/dL (75.63%), respectively. This reduction may be explained by the fact that these uricosuric drugs increased UA excretion (3.55 ± 0.55 mg/dL and 5.95 ± 0.39 mg/dL, respectively).

Spectrophotometric quantification showed that the serum UA concentration decreased in animals treated with LIPO + EREC or LIPO + GOIA, to 5.02 ± 0.93 mg/dL (85.37%) and 10.80 ± 0.85 mg/dL (68.52%), respectively, compared with the hyperuricemic control group (Table 2 and Fig. 4a). Treatment with either liposomal formulation increased UA excretion to 3.34 ± 0.65 mg/dL (LIPO + EREC) and 3.19 ± 0.78 mg/dL (LIPO + GOIA) compared with the negative control group (Fig. 4b and Table 2). Treatment with either liposomal formulation had no effect on XO activity (Table 2). The occurrence of the anti-hyperuricemic effect promoted by the treatment of the rats with the liposomal formulations containing EREC and GOIA may be related to the fact that the in vivo experiments have lasted more than 5 h, indicating that the release of both substances was successful (see Supplementary Fig. S3).

## Discussion

Natural products have already demonstrated promising effects in some disease therapies, such as gout and hyperuricemia^14,15,18^, but their application faces some challenges, such as low solubility and stability in physiological environments, and some toxicity due to the presence of some functional groups, e.g. the α-methylene-γ-lactone group in the case of the sesquiterpene lactones^17,18,23^. Within this context, suitable nanostructured formulations emerge as a promising strategy to improve the therapeutic index of these substances. Liposomal formulations have presented some advantages as drug delivery systems due to their high biocompatibility and the physical compartment of the lipid bilayer: they may encapsulate lipophilic substances, increasing their solubility and stability in physiological environments, and they may reduce the toxicity associated with the substances, due to their ability to promote controlled drug release^21–23^.

Previous studies have demonstrated that nanoformulations are an alternative strategy to maximize the therapeutic value against cancer of a sesquiterpene lactone named parthenolide^24^. A nanoliposomal formulation containing a sesquiterpene lactone named artemisinin presented important activity against visceral leishmaniasis in a murine model^25^.

Thus, in this work, we designed liposomal formulations to encapsulate two bioactive substances from Brazilian flora, the sesquiterpene lactones EREC and GOIA, aiming to reduce their toxicity while maintaining their anti-hyperuricemic activity. Two liposomal formulations (LIPO + EREC and LIPO + GOIA) were developed for oral administration, as a novel approach for treating gout and hyperuricemia. These pathologies, characterized by high UA levels, involve treatment with drugs with adverse effects^5,6,9^.

In our study, the presence of SPC in both formulations served as an important component for liposome formation. Its amphiphilic properties contributed to the self-assembly of lipids into bilayers, creating vesicles that were able to encapsulate the hydrophobic compounds EREC and GOIA^21,22,25^. Furthermore, the formation of lipid vesicles and the existence of an internal aqueous compartment in the developed formulations indicate that it is also possible to co-encapsulate hydrophilic drugs in liposomes. The differences between the molecular structure of EREC and GOIA may lead to specific chemical reactions or interactions within the liposomes.

Characterization of our liposomal formulations revealed favorable properties, including nanometric size and a narrow size distribution (due to the size calibration by extrusion across 100-nm pore size polycarbonate membrane), as well as a slightly negative zeta potential. The PDI presented by our liposomal formulations was lower than the one presented by the nanoliposomal formulation with artemisinin developed by Want et al*.* (2017)^25^. No significant change in mean diameter, PDI and zeta-potential was observed, supporting colloidal stability of the liposome suspension under temperature stress condition. Despite that, the low zeta potential may not guarantee long-term colloidal stability of the formulation under storage. To address this issue, further studies should consider the development of a solid form of the formulations, by freeze-drying the liposomal suspensions in the presence of cryoprotective sugar.

The efficient encapsulation of EREC and GOIA highlighted the potential for sustained therapeutic efficacy. The lipophilic nature and specific functional groups of EREC and GOIA promoted effective encapsulation within the liposomes, resulting in the observed sesquiterpene lactones concentrations and EEs, which can be attributed to the compatibility of EREC and GOIA with the liposomal membrane, driven by their respective molecular structures. The observed EREC and GOIA concentrations in the liposomal formulation reflect the efficiency of the encapsulation process and the loading capacity of the liposomes, indicating a strong affinity, probably due to hydrophobic interactions between the hydrophobic regions of the substances and the liposomal membrane. The EEs values determined for both developed formulations (70–80%) were higher than the one obtained by Want et al*.* (2017), when considering their nanoliposomal formulation with artemisinin (47%)^25^.

We also used HPLC to evaluate our formulations within simulated physiological environments, including gastric fluid without enzymes (pH 1.2), acetate buffer (pH 4.5), and simulated intestinal fluid without enzymes (pH 6.8). The significance of this analysis stems from the intended oral delivery of these formulations, emphasizing the need to understand their behavior under conditions that mimic the gastrointestinal tract. Both developed formulations presented good stability under simulated physiological environments, but the LIPO + EREC showed some instability at the pH 1.2, indicating that liposomal encapsulation of EREC was not able to increase its stability in the acidic medium. The low stability presented by LIPO + EREC may also be related to the half-life of EREC release from liposomes (3.7h), indicating a relatively fast substance release. Furthermore, the stability evaluation over 12 months indicated a small decrease in the EREC concentration in LIPO + EREC, suggesting some degradation, while LIPO + GOIA demonstrated remarkable stability. The observed instability at pH 1.2 and the small decrease in substance concentration over 12 months presented by the LIPO + EREC is probably due to the fact that EREC has a methyl group in the C13 instead of the methylene group (as GOIA). The absence of α-methylene-γ-lactone group to stabilize the molecule by resonance in EREC probably conferred a lower stability, when compared to GOIA^19,20^. Besides that, it is reasonable to consider that the stability evaluation over 12 months, with storage at 4°C, indicated remarkable stability in both liposomal formulations. Previous studies have demonstrated that nanoformulations containing parthenolide or artemisinin were able to increase these substances’ solubility, cellular uptake and stability^24,25^.

Our results emphasize the importance of ongoing monitoring and optimization in formulation development to ensure the sustained efficacy and stability of pharmaceutical products. The results should be considered in the context of the intended oral administration, ensuring that the formulation can deliver the desired therapeutic effect while minimizing toxicity.

In vitro assays with Caco-2 cells revealed that the empty liposomes did not exert any toxicity. However, LIPO + GOIA did significantly reduce cell proliferation, suggesting an antiproliferative effect. Previous studies have explored the antiproliferative activity of a nanoformulation containing the sesquiterpene lactone parthenolide in cancer therapy^24^, characterized by a more effective induction of apoptosis, and have demonstrated the low toxicity of this novel therapeutic agent^24^. Liposomal encapsulation provides a protective barrier that prevents the immediate interaction between cyclic or acyclic methacrylate ester groups with biological molecules and direct contact between the α-methylene-γ-lactone group and cellular components. This strategic encapsulation minimizes the risk of toxicity, ensuring that the therapeutic benefits of the sesquiterpene lactones can be harnessed while maintaining safer and more effective treatments for conditions like gouty arthritis and hyperuricemia. The reduction in cell proliferation produced by LIPO + GOIA indicated lower cytotoxicity of the GOIA formulated, evidencing that GOIA when encapsulated in the liposome did not produce toxicity, while free GOIA was cytotoxic^20^. Furthermore, a reduction in Caco-2 cell proliferation may be associated with changes in UA metabolic pathways, leading to decreased serum UA levels.

In our in vivo assays, both liposomal formulations significantly reduced serum UA levels, comparable to the traditional clinical drugs, in animals with induced hyperuricemia. Importantly, the liposomal formulations increased UA excretion without affecting XO activity, providing a unique mechanism for modulating hyperuricemia. The anti-hyperuricemic activity may be related to the successful release of eremantholide C and goyazensolide from the liposomes during the development of the in vivo assay. In the same manner, a previous study has demonstrated that a formulation with the lactone esculetin was able to reduce UA levels and treat hyperuricemia^26^.

The results obtained from the characterization and the stability evaluation of our developed formulations containing SPC and the sesquiterpene lactones EREC and GOIA are promising for elucidating potential therapeutic interventions for hyperuricemia-related conditions.

## Materials and methods

### Chemicals and reagents

Chloroform was obtained from Synth^®^ (São Paulo, Brazil). Ethanol was obtained from Vetec^®^ (Rio de Janeiro, Brazil). Soybean phosphatidylcholine (SPC, Phospholipon® 90G) was obtained from Lipoid (Ludwigshafen, Germany). High-performance liquid chromatography (HPLC) grade acetonitrile was obtained from J.T. Baker (Mexico City, Mexico). All other chemicals were of analytical grade. The water was ultra-purified by a Milli-Q System (Direct-Q3, Millipore, Darmstadt, Germany).

Dulbecco’s Modified Eagle's Medium (DMEM), fetal bovine serum (FBS), L-glutamine, antibiotics and dimethyl sulfoxide (DMSO) were from Sigma-Aldrich (St. Louis, MO). Sulforhodamine B (SRB) was also from Sigma-Aldrich.

Ketamine (injectable Dopalen) was obtained from Ceva Santé Animale (Libourne, France), and xylazine (injectable Dopaser) was obtained from Hertape Calier (Minas Gerais, Brazil). Xanthine, potassium oxonate, UA, bovine serum albumin (BSA), probenecid, benzbromarone and allopurinol were obtained from Sigma-Aldrich.

### Obtaining sesquiterpene lactones

EREC (colorless solid, ethyl acetate, melting point at 214–215 °C, Fig. 1(1)) was isolated from the ethanolic extract of *Lychnophora* *trichocarpha* as described previously^27,28^. GOIA (white solid, chloroform, melting point at 168.7–169.5 °C, Fig. 1(2)) was isolated from the chloroformic extract of *Lychnophora* *passerina* as described previously^29^.The chemical structures of these substances were elucidated by nuclear magnetic resonance (NMR) data and by comparison with spectral literature data^19,27,30^.

### Eremantholide C (1) (Fig. 1)

6,9-epoxy-2H-1,4-dioxacyclodeca[c,d]pentalene-2,7(4aH)-dioxane, 2a,3,5,6,11a,11b-hexahydro-3-hydroxy-2a,6,10-trimethyl-3-(1'-methylene)2aR,3S,4aR*,6S*,10Z,11aS*,11bS*.

^1^H NMR (CDCl_3_, 400 MHz): 5.63 (s, H-2); 6.03 (m, H-5); 5.02 (m, H-6); 2.82 (dd, *J* 4.3; 7.1 Hz, H-7); 4.10 (ddd, *J* 2.5; 4.3; 12.0 Hz, H-8); 2.47 (dd, *J* 2.5; 13.5 Hz, H-9a); 2.00 (dd, *J* 12.0; 13.5 Hz, H-9b); 1.18 (s, H-13); 1.46 (s, H-14); 2.06 (t, *J* 1.9 Hz, H-15); 5.31 (bs, H-2′a); 5.07 (m, H-2’b); 1.91 (bs, H-3′); 3.79 (s, OH)^19,27^.

^13^C NMR (CDCl_3_, 100 MHz): 205.72 (C-1); 104.52 (C-2); 187.13 (C-3); 130.03 (C-4); 134.71 (C-5); 81.46 (C-6); 62.47 (C-7); 78.38 (C-8); 43.50 (C-9); 90.17 (C-10); 59.85 (C-11); 175.67 (C-12); 21.91 (C-13); 20.49 (C-14); 20.33 (C-15); 106.10 (C-16); 142.12 (C-1′); 115.90 (C-2′); 18.99 (C-3′)^19,27^.

### Goyazensolide (2) (Fig. 1)

(2Z,4R,8R,9S,11R)-2-(Hydroxymethyl)-11-methyl-7-methylene-6,12-dioxo-5,14-dioxatricyclo[9.2.1.04,8]tetradeca-1(13),2-dien-9-yl methacrylate.

^1^H NMR (CDCl_3_, 400 MHz): 5.83 (s, H-2); 6.02 (bs, H-5); 5.34 (m, H-6); 3.80 (m, H-7); 4.53 (dt, *J* 1.85; 11.80 Hz, H-8); 2.52 (dd, *J* 11.80; 13.77 Hz, H-9a); 2.33 (dd, *J* 1.85; 13.77 Hz, H-9b); 6.23 (d, *J* 2.74 Hz, H-13a); 5.49 (d, *J* 2.74 Hz, H-13b); 1.54 (s, H-14); 4.39 (m, H-15a,b); 6.02 (bs, H-3′a); 5.56 (m, H-3’b); 1.83 (s, H-4′)^30^.

^13^C NMR (CDCl_3_, 100 MHz): 204.74 (C-1); 106.65 (C-2); 184.48 (C-3); 134.52 (C-4); 135.32 (C-5); 81.71 (C-6); 50.87 (C-7); 73.34 (C-8); 43.92 (C-9); 89.92 (C-10); 133.15 (C-11); 168.93 (C-12); 124.76 (C-13); 20.71 (C-14); 63.12 (C-15); 166.94 (C-1’); 135.32 (C-2′); 126.69 (C-3′); 17.99 (C-4′)^30^.

NMR data of **1** and **2** (Fig. 1) was assigned with the aid of 2D NMR experiments, including ^1^H–^1^H homonuclear correlation (COSY), ^1^H-^13^C direct (HSQC), and long-range (HMBC) heteronuclear correlations. The chemical shifts, *d*, were expressed in ppm and the coupling constants, *J*, were given in Hz.

### Preparation and characterization of the liposomal formulations

Two liposomal suspensions were prepared from SPC: one containing EREC and one containing GOIA. The liposome formulations of the sesquiterpene lactones were prepared by the ethanol injection method as described previously^31^, with modifications. Briefly, 73 mg of SPC were dissolved in 75 µL of ethanol, the sesquiterpene lactone was added to the lipid solution at 1:20 substance/lipid mass ratio, and the resulting solution was incubated at 40 °C until complete dissolution. The ethanolic lipid solution was then rapidly injected in 0.75 mL of 1× PBS (pH 7.4), using a 1-mL syringe, and the resulting suspension was kept for 15 min at room temperature under magnetic stirring. The liposome size was calibrated by repeated extrusions (10 times) across 100 nm pore size polycarbonate membranes. Finally, the liposome suspension was dialyzed against 1× PBS using a membrane (molecular weight cut-off = 15 kDa) for 4 h at 4 °C to remove ethanol and non-encapsulated substance. Empty (substance-free) liposomes (LIPO) were also prepared by using the same protocol.

The mean hydrodynamic diameter (z-average) and the polydispersity index (PDI) were determined by Dynamic Light Scattering (DLS), using a nanoparticle size analyzer (Zetasizer S90; Malvern Panalytical, Malvern, United Kingdom), after the dilution of the liposome suspensions in 1× PBS (1:100 v/v dilution). The zeta potential (ζ) of the vesicles was also measured by Electrophoretic Light Scattering (ELS), using the same equipment. The sesquiterpene lactones were quantified in the initial (before extrusion) and in the final liposome suspensions, after redissolution of the liposomal formulation in ethanol (1:20 v/v dilution), by UV spectrophotometry (Varian Cary^®^ 50 UV–Vis spectrophotometer, Varian, Palo Alto, CA, USA) at 254 nm, at room temperature, to determine the encapsulation efficiency (EE) and the stability over 12 months after their storage at 4 °C (their content was analyzed after 1 week [i.e., 0 months] and 1, 2, 3, 6, and 12 months). All measurements were performed in triplicates, and the means were calculated.

The formulation encapsulation efficiency (%EE) was determined as:

$$\% \mathrm{EE }= \frac{100 \times \mathrm{ substance\, concentration\, in\, the\, final\, suspension }}{\mathrm{substance \,concentration\, in\, the\, suspension\, before\, dialysis}}$$

## Evaluation of the stability of empty (substance-free) liposome suspension under temperature stress

Three samples of the empty (substance-free) liposome (LIPO) were prepared independently and stored in amber glass vials, protected from light and under argon atmosphere. Following ANVISA guidelines for stability study of cosmetic products^32^, the samples were submitted to temperature stress, with alternate cycles of 24 h at 40 ± 2 °C and 24 h at 4 ± 2 °C, for four weeks. A reference sample was kept for 4 weeks at 25°C, protected from light. At time intervals of 0, 1, 2, 3 and 4 weeks, a vial from each sample was open and evaluated for mean hydrodynamic diameter, PDI and zeta-potential, as described above.

### Evaluation of the encapsulation efficiency of calcein as a fluorescent hydrophilic marker

To evaluate the encapsulation of calcein in the liposome formulations of the sesquiterpene lactones, the formulations were prepared by the ethanol injection method as described above but replacing PBS with 60 mM calcein solution at pH 7.4. The EE of calcein was determined, exploiting the marked fluorescence quenching of calcein in concentrated aqueous solution and the high fluorescence yield in diluted solution^33^. Briefly, the liposome suspensions were diluted 20× in 1× PBS, after the extrusion step and 5 µL of the diluted suspension was added to a 1-cm cuvette containing 2 mL of 1× PBS, and fluorescence intensity was measured on a Cary Eclipse™ spectrofluorometer (Varian Inc., Australia), using excitation and emission wavelengths of 490 mn and 515 nm, respectively, before (Fext) and after (Ftot) addition of 5 µL of 20% (m/v) Triton X-100.

The encapsulation efficiency (%EE) was calculated as:

$$\%\mathrm{EE}= \frac{100 \times ({\text{Ftot}}-{\text{Fext}})}{{\text{Ftot}}}$$

### Quantification of the sesquiterpene lactones in the liposomal formulations and evaluation of their stability under simulated physiological environments by HPLC

The buffer media—simulated gastric fluid without enzymes (pH 1.2), acetate buffer (pH 4.5), and simulated intestinal fluid without enzymes (pH 6.8)—were prepared as described in the United States Pharmacopeia (37th revision)^34^.

The 10× phosphate-buffered saline (PBS) was prepared by adding 82 g of sodium chloride, 10.5 g of disodium phosphate, and 3.55 g of monobasic sodium phosphate into a volumetric flask, bringing the volume to 1 L with Milli-Q water. The pH was adjusted to 7.4 with a pH meter (Digimed model DM-20, Digicron Analitica Ltda, São Paulo, Brazil). The 1× PBS was prepared by diluting 100 mL of 10× PBS in 900 mL of Milli-Q water (pH adjusted to 7.4).

EREC and GOIA that were efficiently encapsulated in liposomes were quantified using an HPLC system (Waters Alliance^®^ e2695, Waters, MA, USA) coupled to an ultraviolet–visible (UV–Vis, Waters 2489, MA, USA) detector. The samples were prepared at room temperature. The chromatographic conditions are described in Table 3. The HPLC methods for the quantification and stability evaluation of the liposomal formulations containing EREC and GOIA under different pH conditions: simulated gastric fluid without enzymes (pH 1.2), acetate buffer (pH 4.5), and simulated intestinal fluid without enzymes (pH 6.8), were adapted from the method developed and validated by Caldeira et al*.* (for EREC)^19^ and Ugoline et al*.*^29^ and Tana et al*.*^20^ (for GOIA). Analytical curves were obtained by EREC and GOIA peaks areas quantified by HPLC at 267 nm. Variations in the EREC and GOIA peak areas were analysed at time zero, 30 min, 60 min, and 7 h. Furthermore, peak purity was evaluated to attest to the absence of coelution of other interfering substances with the chromatographic signal of the sesquiterpene lactones.

**Table 3:** Chromatographic conditions to quantify eremantholide C (EREC) and goyazensolide (GOIA) during the in vitro studies.

Chromatographic conditions	EREC^19^	GOIA^20,29^
Detector	Ultraviolet–Visible	Ultraviolet–Visible
Wavelength (nm)	267	267
Mobile phase	Acetonitrile:water (50:50)	Acidified water 0.01%:acetonitrile (60:40)
Column	C18 (150 × 4.6 mm; 3 µm) Luna^®^—Phenomenex	C18 (150 × 4.6 mm; 5 µm) Agilent Zorbax Eclipse XDB
Temperature (°C)	30	30
Injection volum (µL)	25	20
Flow (mL/min)	1	1
Concentration range (µg/mL)	10.0–50.0	10.0–50.0
Retention time (min)	6.5	3.5

### In vitro release test of liposome-encapsulated substances

In vitro study of EREC and GOIA release from liposomes was carried out with a dialysis device. Immediately after removing the ethanol residue and non-encapsulated drug, the liposome suspensions were divided into sextuplicates of 100 μL and added to Slide-A-Lyzer mini 10 kDa MWCO dialysis devices (Thermo Scientific^®^) coupled to an eppendorf tube containing 1.5 mL of 1× PBS solution under agitation in a ThermoMixer (Eppendorf^®^) at 300 rpm/37 °C. A total of 5 μL of the dialyzed suspension was withdrawn at intervals of 0, 0.5, 1, 2, 3, 4, 5, 6, and 24 h and diluted with 100 μL of EtOH to read the absorbance at 254 nm in a Synergy™ HTX microplate spectrophotometer (BioTek^®^, USA), with replacement of 5 μL of 1× PBS after collection of each sample. Empty (substance-free) liposomes were dialyzed under the same conditions, to be used for background subtraction.

### Cell culture

Caco-2 cells (from American Type Culture Collection, Manassas, VA, USA) were grown in DMEM supplemented with 10% FBS, 1% l-glutamine, and 1% penicillin/streptomycin. The cells were maintained at 37 °C in a humidified atmosphere containing 5% CO_2_. Medium was replaced every 48 h. The cells were trypsinized every 3–5 days and further sub-cultured by splitting them in a ratio of 1:3. Treatments with LIPO and the liposomal formulations containing EREC or GOIA were performed in DMEM supplemented with 10% FBS.

### Cell viability

To evaluate Caco-2 viability following treatment with LIPO and the liposomal formulations containing EREC or GOIA, the sulforhodamine B (SRB) assay was performed as described previously^35^. Cells were seeded in 96-well plates at a density of 9.0 × 10^4^ cells/mL and incubated until the following day to allow adhesion. One plate was stopped at this time (time zero [T0]) to determine the initial number of cells. The other plate (named “treated plate”) was treated with LIPO, the liposomal formulations containing EREC or GOIA at different concentrations, or an equal amount of medium (normal control), as described in Table 4, and incubated for another 24 h (T24).

**Table 4:** Specification of the treatments evaluated during the sulforhodamine B assay.

Treatments	Tested concentrations (µg/mL)
Dulbecco’s Modified Eagle’s Medium, 10% fetal bovine serum, and 1% l-glutamine (normal control)	–
Substance-free liposomes (LIPO)	0.001; 0.01; 0.05; 0.1; 0.5
Liposomal formulation with eremantholide C (LIPO + EREC)	0.001; 0.01; 0.05; 0.1; 0.5; 1.56; 3.125; 6.25; 12.5; 25
Liposomal formulation with goyazensolide (LIPO + GOIA)	0.001; 0.01; 0.05; 0.1; 0.5; 1; 5; 10; 20; 40

After this time, for both the T0 and T24 plates, the medium was removed and after washing three times with 1× PBS, the cultures were fixed with 100 μL of 10% ice-cold trichloroacetic acid (TCA) and incubated at 4 °C for 30 min. From this point, the T0 and T24 plates were processed simultaneously for the SRB assay. Briefly, the plates were stained with 0.4% SRB. After that, 200 μL of 10 mM Tris base was used to reconstitute the dye. The optical density was measured at λ_excitation_ = 488 nm, λ_emission_ = 585 nm, and gain = 75 (BioTek Synergy HTX Reader, BioTek, Winooski, VT, USA).

The following formula was used to obtain the percentage of cell proliferation:

$$\mathrm{Cell Proliferation } \, ({\%}) = \frac{{\text{T}}24\,\mathrm{ Absorbance}}{{\text{T}}0\,\mathrm{ Absorbance}} \times 100$$

The following formula was used to obtain the viability:

$$\mathrm{Vitality } \, ({\%})= 100- (\frac{\mathrm{Normal \, Control \, Absorbance}-{\text{T}}24\mathrm{ Absorbance }}{\mathrm{Normal \, Control \, Absorbance }} \times 100)$$

### Animals

In vivo assays were performed with male Wistar rats (180–280 g) provided by the Federal University of Ouro Preto (UFOP)'s Animal Center (CCA, UFOP, Ouro Preto, Minas Gerais, Brazil). The rats were housed in groups of four, maintained under a 12-h photoperiod and with ad libitum access to water and food. The experimental protocol was approved by UFOP’s Ethical Committee on the Use of Animals (number: 4079140421) and adhered to the National Institutes of Health *Guide* *for* *the* *Care* *and* *Use* *of* *Laboratory* *Animals* (NIH Publication No. 80-23, revised in 1978). Furthermore, the study is reported in accordance with ARRIVE guidelines.

### Evaluation of the anti-hyperuricemic activity of the formulations

The anti-hyperuricemic experimental procedure was described previously^36,37^. Animals were divided in groups of eight (n = 8/each group). To induce hyperuricemia, rats received an intraperitoneal injection of potassium oxonate (0.5 mL; 200 mg/kg) and an oral gavage of UA (1 mL; 1 g/kg). Thirty minutes after hyperuricemia induction, the following treatments were administered intraperitoneally (0.2 mL):

- Group 1, “normal control”: vehicle (DMSO:Tween 80:distilled water [10:10:80]).
- Group 2, “hyperuricemic/negative control”: vehicle.
- Group 3, “positive control for UA quantification assay”: benzbromarone (10 mg/kg).
- Group 4, “positive control for UA quantification assay”: probenecid (50 mg/kg).
- Group 5, “positive control for XO quantification assay”: allopurinol (10 mg/kg).
- Group 6: LIPO + EREC (25 µg/mL).
- Group 7: LIPO + GOIA (40 µg/mL).

Subsequently, the rats were placed individually in metabolic cages and provided with water (100 mL). After 5 h, urine was collected in graduated tubes and stored at − 20 °C for further UA quantification. The rats’ water intake was measured. The rats were euthanized with a combination of ketamine and xylazine (80 and 20 mg/kg, respectively) and blood samples were collected from the abdominal aorta. The samples were then centrifuged at 3000*g* for 15 min at 4 °C; the supernatant (serum) was collected and stored at − 20 °C for further UA quantification. Liver samples were also collected and stored at − 80 °C for further XO quantification.

### UA quantification

UA levels in blood and urine samples were quantified with a colorimetric technique, a diagnostic kit from Bioclin (Minas Gerais, Brazil), following the manufacturer's instructions.

### XO quantification

XO activity was quantified with a method that was described previously^38,39^, with modifications. This spectrophotometric assay monitors UA formation from xanthine. Briefly, liver samples were homogenized in 1× PBS (5 mL; pH 7.4) and centrifuged at 3000*g* (10 min; 4 °C). The lipidic layer was discarded, and the supernatant was centrifuged at 10,000*g* (60 min; 4 °C). After that, 100 µL of liver homogenate was mixed with 1 mM potassium oxonate in 1× PBS (5.4 mL). The mixture was pre-incubated for 15 min (37 °C); then, a xanthine solution (1.2 mL; 250 mM) was added to it so that the reaction could start. At 0 and 30 min, the reaction was stopped by adding HCl (0.5 mL; 0.6 M). Samples were centrifuged at 3000*g* (5 min), and readings were taken with a Varian Cary^®^ 50 UV–Vis spectrophotometer at 295 nm. The total protein content was quantified by spectrophotometer as described previously^39^ using BSA as a standard. The assays were performed in triplicate. The enzyme activity is expressed as nanomoles of UA produced per minute by 1 mg of protein.

### Statistical analysis

Statistical analysis was performed using GraphPad Prism version 6.01 (GraphPad Software, San Diego, CA, USA). The results are expressed as the mean ± standard error of the mean. The data were analyzed with one-way analysis of variance (ANOVA) followed by the Dunnett and Tukey tests. A P-value ≤ 0.05 was considered statistically significant.

## Conclusion

Our study represents a significant advancement in gout and hyperuricemia treatment. We demonstrated that two liposomal formulations containing the sesquiterpene lactones eremantholide C and goyazensolide and designed for oral administration exhibited nanometric size, a narrow size distribution and good stability over 12 months after storage at 4°C and under simulated physiological environments. This strategic approach was able to maintain the anti-hyperuricemic activity exhibited by these free substances: our liposomal formulations were able to reduce uric acid levels in hyperuricemic Wistar rats. Thus, our objectives were achieved: the eremantholide C and goyazensolide liposomal formulations have maintained the anti-hyperuricemic effect, provided stability and solubility and had not shown cytotoxicity. Future studies should consider other parameters involved in the stability of the formulations, such as storage at room temperature and analysis at physiological temperature. Our findings demonstrated that formulations containing eremantholide C and goyazensolide are promising for the treatment of hyperuricemia and chronic gout.

### Supplementary Information

Supplementary Figures.

## Data Availability

All data can be made available by Dênia Antunes Saúde-Guimarães upon request.

## Abbreviations

BSA: Bovine serum albumin
DMEM: Dulbecco's-Modified Eagle's Medium
DMSO: Dimethyl sulfoxide
EE: Encapsulation efficiency
EREC: Eremantholide C
FBS: Fetal bovine serum
GOIA: Goyazensolide
HPLC: High performance liquid chromatography
LIPO: Empty (substance-free) liposome
LIPO + EREC: Liposomal formulation with eremantholide C
LIPO + GOIA: Liposomal formulation with goyazensolide
*L.**passerina*: *Lychnophora* *passerina*
*L.**trichocarpha*: *Lychnophora* *trichocarpha*
NMR: Nuclear magnetic resonance
NSAIDs: Nonsteroidal anti-inflammatory drugs
PBS: Phosphate buffered saline
PDI: Polydispersity index
SPC: Soybean phosphatidylcholine
SRB: Sulforhodamine B
UA: Uric acid
UFOP: Universidade Federal de Ouro Preto
ULDs: Urate-lowering drugs
XO: Xanthine oxidase
ζ: Zeta potential
