# Supplementary material for: Development and characterization of liposomal formulations containing sesquiterpene lactones for the treatment of chronic gout
Source: Sci Rep. 2024 Mar 24;14:6991. doi: 10.1038/s41598-024-57663-1 (PMC10961318; doi:10.1038/s41598-024-57663-1)
Supplement: Supplementary file 1 — Supplementary Figures. [file 41598_2024_57663_MOESM1_ESM.docx]

**Supplementary Material**

**
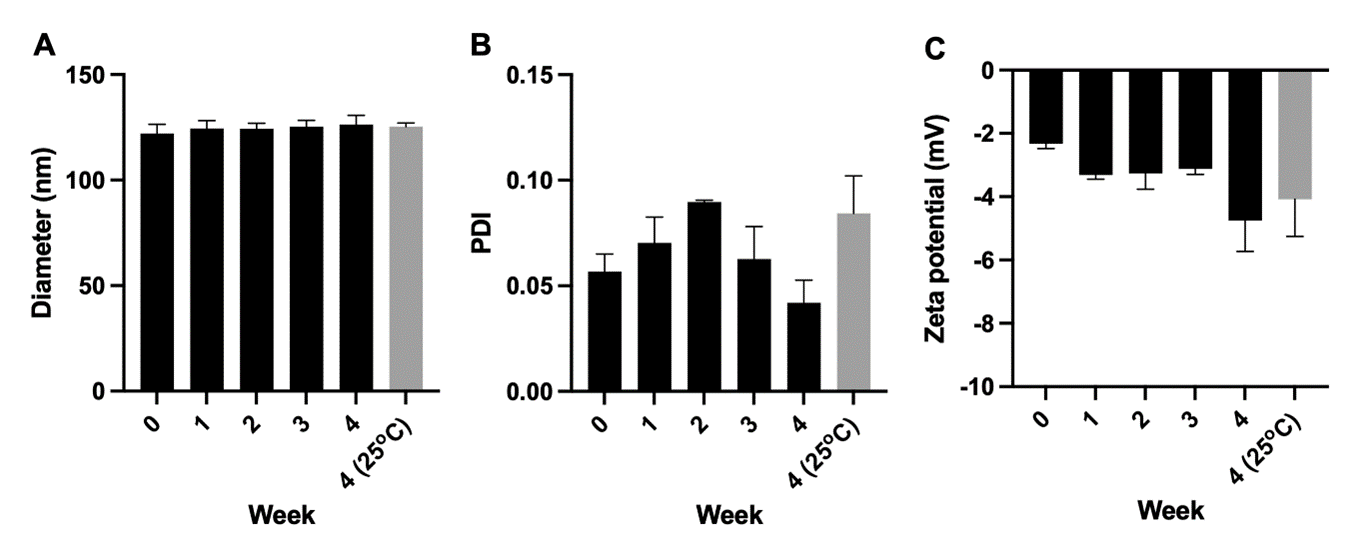
**

**Figure S1.** Stability of empty (substance-free) SPC liposome suspension under temperature stress. Empty SPC liposome suspension (LIPO) was submitted to temperature stress, with alternate cycles of 24h at 40 ± 2°C, and 24h at 4 ± 2°C - for four weeks. Mean hydrodynamic diameter (A), PDI (B) and zeta potential (C) were evaluated at time zero and on each week. A reference sample was kept for 4 weeks at 25°C. Values were expressed as mean ± S.E.M. (n= 3 samples prepared independently). Repeated measures One-Way ANOVA was used for statistical significance. Results show that no significant change in mean diameter, PDI and zeta-potential was observed, supporting colloidal stability of the liposome suspension under temperature stress condition.





**Figure S2.** Encapsulation efficiency of calcein, as a fluorescent hydrophilic marker of internal aqueous compartment, in liposomal suspensions incorporating EREC, GOIA or no substance. LIPO = substance-free SPC liposomes; LIPO + EREC = liposomal SPC formulation containing eremantholide C; LIPO + GOIA = liposomal SPC formulation containing goyazensolide. Values are expressed as mean ± S.D. (n= 3 independent formulations). One-Way ANOVA was used followed by Dunnett's test for statistical significance. The differences between LIPO + GOIA and the two other liposomal formulations are statistically significant. The results also show the existence of an internal aqueous compartment, consistent with the formation of lipid vesicles, indicating that it is possible to co-encapsulate an hydrophilic drug in liposomes.

**

**

**Figure S3.** Release kinetics of eremantholide C (EREC) and goyazensolide (GOIA) from the LIPO + EREC and LIPO + GOIA, respectively, at 37°C. LIPO + EREC = liposomal SPC formulation containing eremantholide C; LIPO + GOIA = liposomal SPC formulation containing goyazensolide. *In vitro* release test was evaluated at 37^o^C using a dialysis device. Values are expressed as mean ± S.E.M. (n= 6). The half-life of release was determined after fitting the data with monoexponential decay; the values appear on the graph. The results show that the release of goyazensolide from the formulation is slower than the release of eremantholide C.
